# Irregular work schedule and sleep disturbance in occupational drivers—A nationwide cross-sectional study

**DOI:** 10.1371/journal.pone.0207154

**Published:** 2018-11-15

**Authors:** Inchul Jeong, Jae Bum Park, Kyung-Jong Lee, Jong-Uk Won, Jaehoon Roh, Jin-Ha Yoon

**Affiliations:** 1 Department of Occupational and Environmental Medicine, Ajou University School of Medicine, Suwon, Korea; 2 Department of Preventive Medicine, Institute for Occupational Health, and Graduate School of Public Health, Yonsei University College of Medicine, Seoul, Korea; 3 Incheon Workers’ Health Center, Incheon, Korea; Peking University, CHINA

## Abstract

**Background:**

The objective of this study was to investigate the relationship between irregular work schedules and sleep disturbance and compare the impacts of work schedule on sleep disturbance between occupational drivers and office workers.

**Methods:**

Using data from the 3^rd^ and 4^th^ Korean Working Conditions Survey, 3,070 occupational drivers and 9,898 office workers were included in this study. The subjects’ days of night work, evening work, and subjective complaints of sleep disturbance were investigated along with other covariates.

**Results:**

In the multivariate logistic regression analyses, occupational drivers (odds ratio [OR], 95% confidence interval [CI]: 1.51, 1.11–2.05), workers who were engaged in more night work (2.49, 1.84–3.38 for 1–15 days, and 3.80, 2.67–5.41 for 16–30 days) and evening work (2.22, 1.66–2.97 for 1–15 days, and 1.76, 1.26–2.45) were more likely to report sleep disturbance. Moreover, occupational driving showed significant interaction effects with both night and evening work on sleep disturbance, and therefore, showed higher ORs for sleep disturbance in the 16–30 days night (5.38, 3.40–8.52) and evening (3.13, 1.97–4.98) compared to no night and evening working office workers.

**Conclusions:**

Occupational drivers who are exposed to night work and evening work are at higher risks for sleep disturbance. Therefore, for the public and drivers’ safety, optimal work schedules for minimising sleep disturbance should be developed.

## Introduction

Sleep problems are commonly reported work-related health outcomes,[1] and negatively affect workers in various ways. The negative impacts of sleep problems include loss of productivity, absenteeism, and increased risk for accidents at work.[2] The impacts may differ by occupation or industry; however, sleep problems may lead to fatal accidents in occupations that require a high level of vigilance, such as occupational driving.[3]

Sleepiness at the wheel or microsleeping during work, which are consequences of poor sleep at night,[4] have been frequently reported as important causes of road traffic accidents, since it impairs vigilance and performance while driving.[5–9] Therefore, occupational drivers are required to maintain alertness during work, since their alertness is closely related to the public safety, as well as the safety of drivers themselves. Nevertheless, it has been reported that sleep problems are prevalent among occupational drivers.[10, 11] Thus, it is important to investigate the factors related to sleep problems among occupational drivers.

A number of previous studies have reported on sleep problems among drivers. In these studies, it was reported that sleep-related factors such as obstructive sleep apnoea syndrome, sleep hours, and quality of sleep; work-related factors such as night driving, shift work, and longer period for driving[12–17] were related to sleepiness at the wheel or accidents. However, in contrast to the relationship between work schedule and sleep disorder which is well-known in general working population,[18, 19] only a few studies have examined the relationship between work schedule and sleep problems in occupational drivers.[17, 20] Moreover, there is a lack of studies examining whether there is a difference in the impact of work schedule on sleep problems between occupational drivers and other workers.

According to the Organisation for Economic Co-operation and Development (OECD) statistics, Korea is one of the countries with the highest rates of fatalities due to traffic accidents, being the highest among OECD countries in 2012, and fourth in 2015.[21] Therefore, research on factors related to sleep disturbance in occupational drivers are necessary in Korea. However, to the best of our knowledge, no such studies have been conducted targeting Korean drivers.

Therefore, the objective of this study was to investigate the relationship between irregular work schedule, such as night and evening work, and sleep disturbance. Moreover, this study aimed to compare the impact of work schedule on sleep disturbance between occupational drivers and office workers.

## Materials and methods

### Study subjects

In this study, data from the third and fourth Korean Working Conditions Survey (KWCS) were used, which were conducted by the Korea Occupational Safety and Health Agency in 2011 and 2014, respectively. The study population for the KWCS was economically active workers aged ≥15 years, and participants were selected using multi-stage random sampling method based on the Population and Housing Census.[22] Among the total study population of 100,039 (50,032 in the third survey and 50,007 in the fourth survey), according to the Korean Standard Classification of Occupations, 3,070 occupational drivers such as taxi drivers, bus drivers, and truck drivers, and 9,898 office workers who responded that they are not exposed to noise and vibration during work were included in this study, since occupational drivers are exposed to noise and vibration during work, which have been reported as risk factors for sleep disturbance in previous studies.[23]

### Assessment of work schedule and sleep disturbance

Evening work was defined as working at least two hours from 18:00 to 21:59, and night work was defined as working at least two hours from 22:00 to 05:59, according to Korean Labor Standards Act, Article 56 (https://elaw.klri.re.kr). The participants were asked about their night and evening working days per month. According to the response, the participants were categorised into three groups: none, 1–15 days, 16–30 days working groups, both for night and evening work. Sleep disturbance was investigated by a subjective question of reporting insomnia or sleep disorder in the recent 12 months.

### Covariates

Sex, age, education level, marital status, working hours, and job-related stress were used as covariates in this study. Age was divided into three groups: 20–39, 40–59, and ≥60. Education level was divided into three groups based on the high school education: less than high school graduation, high school graduation, and college or above. Marital status was divided into two groups of yes and no. Working hours of the subjects were divided into three groups based on the Labor Standards Act of Korea. In Korea, working 40 hours per week is regarded as standard work, and extended work of less than 12 hours per week is allowed. Therefore, it was divided into <40 hours/week, 40–51 hours/week, and ≥52 hours/week. Job-related stress was investigated by a subjective question of suffering from stress during work, and five choices were given: always, mostly, sometimes, rarely, never. Subjects whose responses were the former three were categorised into the “yes” group, and the latter two were categorised into the “no” group.

### Statistical analysis

χ^2^-tests and logistic regression analyses were conducted to compare the characteristics of subjects according to the presence of sleep disturbance. In the logistic regression analyses, odds ratios (ORs) and 95% confidence intervals (CIs) for reporting sleep disturbance were estimated, and linear trend tests were also conducted. The logistic regression analyses were conducted with two models: model 1 (crude model), and model 2 (adjusted for sex, age, education level, marital status, working hours, and job-related stress). All statistical tests were two-tailed, and p-values less than 0.05 were regarded as statistically significant. All statistical analyses were conducted with the SAS software package version 9.4 (SAS Institute Inc., Cary, NC, USA).

### Ethics statement

Written informed consent was obtained from all participants, and the Institutional Review Board of the Yonsei University Health System approved this study (Y-2017-0035).

## Results

The prevalence of night work was almost five times higher in occupational drivers compared to office workers [1,308 (42.6%) in occupational drivers, and 775 (7.8%) in office workers, p<0.001]. The prevalence of evening work was also higher in occupational drivers [756 (67.5%) in occupational drivers, and 3,506 (35.4%) in office workers, p<0.001].

The proportions of workers who reported sleep disturbance were 3.5% (106 out of 3,070) among occupational drivers and 2.1% (212 out of 9,686) in office workers. Among occupational drivers, distributions of sex, age, job-related stress, night work, and evening work were significantly different according to sleep disturbance status with females, subjects with age of 40–59 years, job-related stress, and 16–30 days of night and evening work being more likely to report sleep disturbance in the univariate analyses. On the other hand, among office workers, distributions of age, education level, job-related stress, night work, and evening work were significantly different, with subjects with age of 60 years and older, education level of less than high school, job-related stress, 1–15 days of night and evening work being more likely to report sleep disturbance (Table 1).

**Table 1:** General characteristics of the study subjects by presence of sleep disturbance.

	Sleep Disturbance									
	Occupational Drivers (N = 3,070)					Office Workers (N = 9,898)				
	Yes		No		p-value	Yes		No		p-value
	n	(%)	n	(%)			n	(%)	n		(%)
Total	106	(3.5)	2,964	(96.6)		212	(2.1)	9,686	(97.9)	
Sex										
Male	99	(3.3)	2,882	(96.7)	0.032	92	(2.0)	4,482	(98.0)	0.446
Female	7	(7.9)	82	(92.1)		120	(2.3)	5,204	(97.8)	
Age, years										
20–39	5	(1.1)	444	(98.9)	0.004	76	(1.9)	3,975	(98.1)	0.035
40–59	85	(4.2)	1,958	(95.8)		110	(2.2)	4,958	(97.8)	
≥60	16	(2.8)	562	(97.2)		26	(3.3)	753	(96.7)	
Education level										
Lower than high school	17	(3.0)	557	(97.0)	0.727	28	(3.5)	773	(96.5)	0.011
High school	72	(3.5)	1,982	(96.5)		76	(2.3)	3,301	(97.8)	
College or above	17	(3.9)	425	(96.2)		108	(1.9)	5,612	(98.1)	
Marital status										
Yes	86	(3.4)	2,480	(96.7)	0.576	163	(2.0)	7,834	(98.0)	0.170
No	20	(4.0)	484	(96.0)		49	(2.6)	1,852	(97.4)	
Working hours/week										
<40 hours	7	(2.8)	248	(97.3)	0.110	9	(1.7)	533	(98.3)	0.675
40–51 hours	41	(2.9)	1,400	(97.2)		124	(2.2)	5,468	(97.8)	
≥52 hours	58	(4.2)	1,316	(95.8)		79	(2.1)	3,685	(97.9)	
Job-related stress										
Yes	93	(3.8)	2,352	(96.2)	0.047	188	(2.6)	7,168	(97.4)	<0.001
No	13	(2.1)	612	(97.9)		24	(0.9)	2,518	(99.1)	
Night work/month										
None	30	(1.7)	1,732	(98.3)	<0.001	175	(1.9)	8,948	(98.1)	<0.001
1–15 days	46	(4.5)	968	(95.5)		20	(5.3)	357	(94.7)	
16–30 days	30	(10.2)	264	(89.8)		17	(4.3)	381	(95.7)	
Evening work/month										
None	12	(1.2)	986	(98.8)	<0.001	75	(1.6)	4,511	(98.4)	<0.001
1–15 days	51	(3.9)	1,265	(96.1)		63	(3.5)	1,743	(96.5)	
16–30 days	43	(5.7)	713	(94.3)		74	(2.1)	3,432	(97.9)	

The percentages mean prevalence of each stratum, and p values were estimated by χ^2^ test.

**Fig 1:**
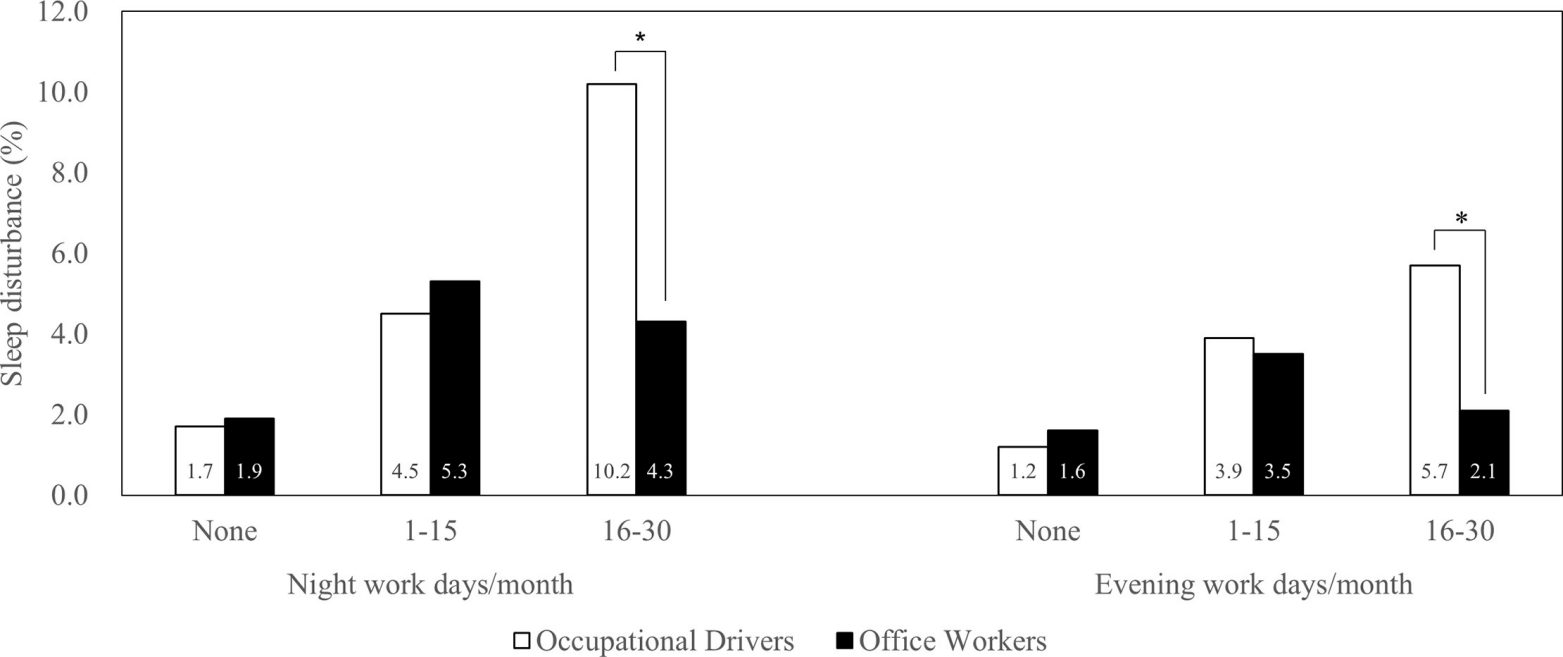
Comparison of sleep disturbance prevalence (%) by occupation and work schedule (* p<0.05 by χ^2^ test between occupational drivers and office workers).

Fig 1 shows the comparison of sleep disturbance prevalence of the two occupation groups by work schedule. The prevalence of sleep disturbance among occupational drivers was significantly higher in the group with 16–30 days of both night and evening work (10.2% and 4.3% with night work; 5.7% and 2.1% with evening work in occupational drivers and office workers, respectively) compared to that of office workers. On the contrary, there was no significant difference between the workers with no night and evening work (1.7% and 1.9% with no night work; 1.2% and 1.6% with no evening work in occupational drivers and office workers, respectively); and 1–15 days of night and evening work (4.5% and 5.3% with night work; 3.9% and 3.5% with evening work in occupational drivers and office workers, respectively).

In the multivariate logistic regression analyses, occupational drivers were more likely to report sleep disturbance (OR, 1.51, 95% CI: 1.11–2.05) after adjustment for sex, age, education level, marital status, working hours, and job-related stress. Additionally, night work and evening work were significantly related to sleep disturbance after adjustment with ORs of 2.49 (1.84–3.38) in the 1–15 days of night working group, and 3.80 (2.67–5.41) in the 16–30 days of night working group; 2.22 (1.66–2.97) in the 1–15 days of evening working group, and 1.76 (1.26–2.45) in the 16–30 days of evening working group, compared to the groups with no night and evening work, respectively. Moreover, the results also showed dose-response relationships (p for trend<0.001 both for night and evening work, Table 2).

**Table 2:** Adjusted odds ratios for sleep disturbance by occupation and work schedule.

	Model 1		Model 2	
	OR	(95% CI)	OR	(95% CI)
Occupation				
Office workers	1.00	Reference	1.00	Reference
Occupational drivers	1.63	(1.29–2.07)	1.51	(1.11–2.05)
Night work/month				
None	1.00	Reference	1.00	Reference
1–15 days	2.60	(1.96–3.45)	2.49	(1.84–3.38)
16–30 days	3.80	(2.74–5.26)	3.80	(2.67–5.41)
Evening work/month				
None	1.00	Reference	1.00	Reference
1–15 days	2.40	(1.81–3.18)	2.22	(1.66–2.97)
16–30 days	1.78	(1.35–2.36)	1.76	(1.26–2.45)

Model 1: Crude model

Model 2: Adjusted for sex, age, education level, marital status, working hours, and job-related stress

OR, odds ratio; CI, confidence interval

When the analyses were conducted only with occupational drivers, more strong and linear relationship was shown (Table 3). The ORs for sleep disturbance were 2.62 (1.62–4.23) in the 1–15 days of night working group, and 5.82 (3.37–10.06) in the 16–30 days of night working group, compared to the no night working group. Additionally, the ORs were 3.16 (1.65–6.04) in the 1–15 days of evening working group, and 4.42 (2.24–8.70) in the 16–30 days of evening working group, compared to the no evening working group. Furthermore, trend tests for both night and evening work were also significant (p for trend <0.001).

**Table 3:** Adjusted odds ratios for sleep disturbance by work schedule in occupational drivers.

	Model 1		Model 2	
	OR	(95% CI)	OR	(95% CI)
Night work/month				
None	1.00	Reference	1.00	Reference
1–15 days	2.74	(1.72–4.38)	2.62	(1.62–4.23)
16–30 days	6.56	(3.89–11.06)	5.82	(3.37–10.06)
Evening work/month				
None	1.00	Reference	1.00	Reference
1–15 days	3.31	(1.76–6.25)	3.16	(1.65–6.04)
16–30 days	4.96	(2.59–9.47)	4.42	(2.24–8.70)

Model 1: Crude model

Model 2: Adjusted for sex, age, education level, marital status, working hours, and job-related stress

OR, odds ratio; CI, confidence interval

Table 4 demonstrates the interaction effect of occupational driving and work schedule on sleep disturbance. The OR was not higher in none night working (0.87, 0.56–1.35) and in none evening working (0.87, 0.56–1.35) occupational drivers than in none of them office workers. When compared to the reference group of no night working office workers, the OR was higher in the 1–15 days of night working office workers (2.87, 1.78–4.63) than in 16–30 days night working office workers (2.33, 1.37–3.96). On the other hand, the OR was higher among the 16–30 days of night working drivers (5.38, 3.40–8.52) than 1–15 days night working drivers (2.26, 1.52–3.35). The result also showed significant interaction effect (p for interaction = 0.027). The interaction effect of occupational driving and evening work also showed significant result (p for interaction = 0.001). When compared to no evening working office workers, the OR was significantly higher in the 1–15 days of evening working office workers (2.13, 1.51–3.00), and in occupational drivers, the OR increased with the days of evening work (2.10, 1.36–3.24 in 1–15 days of evening working drivers; 3.13, 1.97–4.98 in the 16–30 days of evening working drivers). However, the difference was not significant in the 16–30 days of evening working office workers.

**Table 4:** Interaction effect of occupational driving and work schedule on sleep disturbance.

	Office workers		Occupational drivers		p-value for interaction
	OR	(95% CI)	OR	(95% CI)		
Night work/month					0.027
None	1.00	Reference	0.87	(0.56–1.35)	
1–15 days	2.87	(1.78–4.63)	2.26	(1.52–3.35)	
16–30 days	2.33	(1.37–3.96)	5.38	(3.40–8.52)	
Evening work/month					0.001
None	1.00	Reference	0.69	(0.36–1.32)	
1–15 days	2.13	(1.51–3.00)	2.10	(1.36–3.24)	
16–30 days	1.22	(0.83–1.79)	3.13	(1.97–4.98)	

Adjusted for sex, age, education level, marital status, working hours, and job-related stress

OR, odds ratio; CI, confidence interval

## Discussion

In this study, sleep disturbance in occupational drivers compared to office workers, and according to work schedule were examined. Also, the interaction effect of work schedule and occupation was examined. The results showed that occupational drivers were more likely to report sleep disturbance compared to office workers, and engaged days of night and evening work were significantly related to sleep disturbance. Furthermore, occupational driving and 16 or more days of night or evening work showed a synergistic effect on sleep disturbance. This is the first large-scale epidemiologic study which examined the interaction effect of occupation and work schedule on sleep disturbance in Korea.

Insomnia, which is included in the definition of sleep disturbance in this study, is a condition, the development of which is reportedly related to a lot of psychological, behavioural, and physiological statuses. The results of this study can be explained by the relationship between the occupational risk factors of drivers and insomnia or its pathway of development. One possible pathway of insomnia development is the inappropriate activation of the central nervous system, also known as hyperarousal.[24] For example, insomnia often precedes clinical post-traumatic stress disorder.[25] This association can be explained by hyperarousal after traumatic events including car accidents.[26] Several studies on neuroendocrine and electrophysiological, as well as neuroimages support the hyperarousal insomnia phenomenon.[27] Driving naturally involves the risk of accidents; therefore, occupational drivers are required to maintain alertness during work, which is closely related to hyperarousal. Alertness is more required during night time driving; hence night time driving may increase the hyperarousal status. In the present study, night time work was more seriously related to sleep disturbance in drivers compared to office workers. This result supported the finding that sleep disturbance during night time work is related to hyperarousal insomnia.

Occupational drivers are also exposed to other several factors that are related to the development of insomnia. First, they are exposed to noise and vibration during work, which are related to activation of the sympathetic nervous system.[28, 29] Therefore, exposure to noise and vibration possibly contributed to the development of insomnia. Previous studies reported that daytime noise is related to subsequent night sleep disturbance,[30, 31] and exposure to noise and vibration is also related to sleep disturbance,[23, 32] which are consistent with the results of this study. Second, job stress, characterised by high demand and low control, is a significant health risk among drivers.[33–35] Also, it was reported that daytime noise exposure can be a source of stress.[36] Moreover, irregular-shift working drivers showed higher stress response in a previous study.[37] Therefore, along with the job stress as a risk factor for sleep disorder,[30, 38] night work-induced stress response could be a plausible explanation for the result of this study. Third, the prevalent use of caffeine in occupational drivers was reported as a countermeasure for sleepiness.[17, 39] Although caffeine use is an effective way of reducing the risk of accidents,[40, 41] its excessive use is also related to sleep disturbance,[42] due to its action as a central nervous system stimulant. Additionally, although the outcome is different from this study, increased cardiovascular disease risk among drivers found in previous studies[43, 44] supports the possibility of hyperarousal in occupational drivers with sleep disturbance.

In the present study, the female occupational drivers had greater prevalence of sleep disturbance compared to male occupational drivers. Traditional family roles are still more dependent on females in the Korean society. Hence, there may be a lack of sleep hours for female drivers even during their resting time. However, female occupational drivers also showed greater prevalence of sleep disturbance compared to female office workers (p<0.001). Furthermore, there were no gender differences in sleep disturbance prevalence in office workers. This suggests that gender plays different roles in sleep disturbance between drivers and office workers. Although we have no information to clarify the cause of such gender difference, the female drivers were the most vulnerable group in the current study. Therefore, more comprehensive studies are needed to prevent sleep disturbance in female drivers.

In the present study, the prevalence of night work was almost five times higher in occupational drivers compared to office workers [1,308 (42.6%) in occupational drivers, and 775 (7.8%) in office workers, p<0.001]. The prevalence of evening work was also higher in occupational drivers [756 (67.5%) in occupational drivers, and 3,506 (35.4%) in office workers, p<0.001]. As we discussed hyperarousal during night time driving, we hypothesised that the effect of night work has a stronger harmful effect on sleep quality in drivers compared to office workers. To clarify our hypothesis, we analysed the interaction effect of night work and driving on sleep disturbance. There were synergistic effects of night work and occupational driving with the greatest odds of sleep disturbance even compared to night working office workers. Therefore, night work schedule should be managed carefully for occupational drivers.

There are certain strengths in this study. First, this is a large-scale epidemiologic study including more than 10,000 workers. Also, since the workers were systematically sampled, the results of this study can be regarded as being representative of Korean drivers. Furthermore, unlike previous studies, which mostly examined the effects of working hours, work schedule, or occupation separately, the effects of work schedule and occupation were examined simultaneously; therefore, the interaction effect could be examined in this study.

However, there are also several limitations when interpreting the results of this study. First, sleep disturbance was investigated based on a single subjective question, not by an objective measure. However, self-reported disturbed sleep was reported as a predictor of accidental death at work.[45] Therefore, answers to the subjective question could be regarded as reliable. Second, due to the cross-sectional design of the study, the direction of the causal relationship is not clear. Although it is not likely that there is a reverse causation in this study, longitudinal studies clarifying the direction of the causal relationship are needed. Finally, it is not clear which occupational risk factors of the drivers interacted with work schedule in this study, which requires further investigation. The relatively low OR values of officer were observed in 16–30 group compare to those of 1–15 group. We further analysed levels of age and work-period according to night and evening work group. The more than 16 days per month group of office have older age and longer work period compare to none and 1~15 days per month group of them, but occupational driver did not (S1 Table). Those suggested that there was heathy worker survival effect in officer, but not in occupational driver. Further studies were needed to clarify those effect in officer, but those studies are beyond aim of current study.

## Conclusions

In conclusion, occupational drivers who are exposed to night work and evening work are at higher risks for sleep disturbance. Therefore, for the safety of the public, as well as the drivers themselves, work schedules for occupational drivers should be adjusted if there are any complaints of sleep disturbance. Moreover, optimal work schedule for minimising sleep disturbance should be developed.

## Supporting information

S1 Table Age and work-period according to night and evening working group. (DOCX)
