# Supplementary material for: Irregular work schedule and sleep disturbance in occupational drivers—A nationwide cross-sectional study
Source: PLoS One. 2018 Nov 15;13(11):e0207154. doi: 10.1371/journal.pone.0207154 (PMC6237347; doi:10.1371/journal.pone.0207154)
Supplement: S1 Table — (DOCX) [file pone.0207154.s001.docx]

Supplementary Table 1 age and work-period according to night and evening working group

|  |  |  | Suffering from sleep disturbance | | | | | | | | | | | | |  |  |
| --- | --- | --- | --- | --- | --- | --- | --- | --- | --- | --- | --- | --- | --- | --- | --- | --- | --- |
|  |  | Occupational Driver | | | | | | |  | Officer | | | | | |  |  |
|  |  |  |  | Yes |  |  | No |  |  |  | Yes |  |  | No |  |  | |
|  |  |  | N | Mean | SD | N | Mean | SD |  | N | Mean | SD | N | Mean | SD |  |  |
| Age (years) | Night work | 0 | 30 | 53.5 | 8.1 | 1732 | 50.0 | 10.7 |  | 175 | 44.5 | 12.8 | 8948 | 42.5 | 11.3 |  |  |
|  |  | 1~15 | 46 | 52.1 | 7.5 | 968 | 52.0 | 9.5 |  | 20 | 39.8 | 7.6 | 357 | 41.9 | 10.8 |  |  |
|  |  | 16~30 | 30 | 48.4 | 8.1 | 264 | 50.6 | 9.2 |  | 17 | 51.8 | 12.1 | 381 | 48.2 | 12.1 |  |  |
|  | Evening work | 0 | 12 | 55.4 | 9.1 | 986 | 50.0 | 10.8 |  | 75 | 44.7 | 11.0 | 4511 | 41.6 | 10.7 |  |  |
|  |  | 1~15 | 51 | 51.5 | 7.2 | 1265 | 51.2 | 10.0 |  | 63 | 38.5 | 10.3 | 1743 | 40.4 | 10.2 |  |  |
|  |  | 16~30 | 43 | 50.3 | 8.5 | 713 | 50.9 | 9.7 |  | 74 | 49.8 | 13.7 | 3432 | 45.4 | 12.3 |  |  |
| Work-period (month) | Night work | 0 | 29 | 168.1 | 128.0 | 1723 | 120.3 | 105.4 |  | 175 | 109.8 | 106.3 | 8894 | 93.9 | 91.9 |  |  |
|  |  | 1~15 | 46 | 132.5 | 105.5 | 960 | 131.5 | 101.8 |  | 20 | 71.1 | 72.7 | 356 | 91.0 | 85.7 |  |  |
|  |  | 16~30 | 30 | 76.0 | 58.4 | 263 | 112.6 | 90.6 |  | 17 | 124.9 | 94.2 | 381 | 102.0 | 95.4 |  |  |
|  | Evening work | 0 | 12 | 202.0 | 143.4 | 979 | 116.9 | 105.0 |  | 75 | 116.1 | 104.9 | 4473 | 97.4 | 93.6 |  |  |
|  |  | 1~15 | 50 | 122.4 | 101.6 | 1259 | 130.8 | 102.9 |  | 63 | 68.1 | 66.5 | 1727 | 95.4 | 87.9 |  |  |
|  |  | 16~30 | 43 | 109.5 | 94.2 | 708 | 118.5 | 100.3 |  | 74 | 131.9 | 117.3 | 3431 | 89.0 | 91.2 |  |  |
